# Tau filaments from amyotrophic lateral sclerosis/parkinsonism-dementia complex adopt the CTE fold

**DOI:** 10.1073/pnas.2306767120

**Published:** 2023-12-15

**Authors:** Chao Qi, Bert M. Verheijen, Yasumasa Kokubo, Yang Shi, Stephan Tetter, Alexey G. Murzin, Asa Nakahara, Satoru Morimoto, Marc Vermulst, Ryogen Sasaki, Eleonora Aronica, Yoshifumi Hirokawa, Kiyomitsu Oyanagi, Akiyoshi Kakita, Benjamin Ryskeldi-Falcon, Mari Yoshida, Masato Hasegawa, Sjors H. W. Scheres, Michel Goedert

**Affiliations:** ^a^Medical Research Council, Laboratory of Molecular Biology, Cambridge CB2 0QH, United Kingdom; ^b^Leonard Davis School of Gerontology, University of Southern California, Los Angeles, CA 90089; ^c^Graduate School of Regional Innovation Studies, Mie University, Tsu 514-8507, Japan; ^d^Department of Pathology, Brain Research Institute, Niigata University, Niigata 951-8585, Japan; ^e^Department of Oncologic Pathology, Graduate School of Medicine, Mie University, Tsu 514-8507, Japan; ^f^Department of Nursing, Suzuka University of Medical Science, Suzuka 513-8670, Japan; ^g^Department of Neuropathology, Amsterdam University Medical Centers (UMC), University of Amsterdam, Amsterdam Neuroscience, Amsterdam 1105 AZ, The Netherlands; ^h^Department of Brain Disease Research, Shinshu University School of Medicine, Matsumoto 390-8621, Japan; ^i^Department of Neuropathology, Institute for Medical Science of Aging, Aichi Medical University, Nagakute 480-1195, Japan; ^j^Department of Brain and Neuroscience, Tokyo Metropolitan Institute of Medical Science, Tokyo 156-8506, Japan

**Keywords:** neurodegenerative disease, amyotrophic lateral sclerosis-parkinsonism dementia complex (ALS/PDC), tau filament assembly, chronic traumatic encephalopathy tau fold

## Abstract

The amyotrophic lateral sclerosis/parkinsonism-dementia complex (ALS/PDC) of the island of Guam and the Kii peninsula of Japan is a fatal neurodegenerative disease of unknown cause that is characterized by the presence of abundant filamentous tau inclusions in brains and spinal cords. Here, we used electron cryo-microscopy to determine the structures of tau filaments from the cerebral cortex of three cases of ALS/PDC from Guam and eight cases from Kii, as well as from the spinal cord of two of the Guam cases. Tau filaments had the chronic traumatic encephalopathy (CTE) fold, with variable amounts of Type I and Type II filaments. Paired helical tau filaments were also found in three Kii cases and tau filaments with the corticobasal degeneration fold in one Kii case. We identified a new Type III CTE tau filament, where protofilaments pack against each other in an antiparallel fashion. ALS/PDC is the third known tauopathy with CTE-type filaments and abundant tau inclusions in cortical layers II/III, the others being CTE and subacute sclerosing panencephalitis. Because these tauopathies are believed to have environmental causes, our findings support the hypothesis that ALS/PDC is caused by exogenous factors.

## Significance

A neurodegenerative disease of unknown cause on the island of Guam and the Kii peninsula of Japan has been widely studied, because patients can suffer from the combined symptoms of motor neuron disease, parkinsonism, and dementia. Abnormal filamentous inclusions made of tau protein characterize this amyotrophic lateral sclerosis/parkinsonism-dementia complex (ALS/PDC) and their formation closely correlates with neurodegeneration. Here, we have used electron cryo-microscopy to show that tau filaments from ALS/PDC are identical to those from chronic traumatic encephalopathy (CTE), a disease caused by repetitive head impacts or blast waves. CTE tau filaments are also found in subacute sclerosing panencephalitis, which is a rare consequence of measles infection. ALS/PDC may therefore also be caused by environmental factors.

Amyotrophic lateral sclerosis/parkinsonism-dementia complex (ALS/PDC or lytico-bodig) is a fatal disease found in the Chamorro population of Guam (1–4), some families on the Kii peninsula of Japan (5, 6), and the Auyu and Jakai people of New Guinea (7). Abundant tau inclusions are present in nerve cells in brains and spinal cords (6, 8, 9) and are enriched in cortical layers II/III (10, 11). Tau inclusions are also found in some glial cells (12). They consist of amyloid filaments that are made of all six brain tau isoforms in a hyperphosphorylated state (8, 13). More variably, assembled Aβ, α-synuclein, and TDP-43 accumulate too (11, 14, 15).

The cause of ALS/PDC is unknown, but it is not a simple genetic disorder in an island-bound geographic isolate (16–18). Exogenous factors may play a role in disease aetiology and pathogenesis, supported by the finding that migrants from the Philippines can develop ALS/PDC after living on Guam for more than two decades (19). With increased Westernisation, the incidence of ALS/PDC is decreasing (20). High prevalence of a retinopathy, called linear retinal pigment epitheliopathy (LRPE), has been reported in Guam and Kii ALS/PDC (21, 22), similar to infestation by a migrating parasite larva. Both disorders have declined in parallel, suggesting a possible link between ALS/PDC and LRPE.

Tau filaments made of all six brain isoforms in a hyperphosphorylated state are also found in Alzheimer’s disease (AD) and in chronic traumatic encephalopathy (CTE) (23, 24). They are found predominantly in cortical layers V/VI in AD (25) and in layers II/III in CTE (26). The latter is caused by repetitive head impacts or exposure to blast waves (27). By cryo-EM, we have shown that tau filaments from AD and CTE each consist of two identical C-shaped protofilaments that comprise residues 306 to 378 (in the numbering of the 441 amino acid tau isoform) (28–30). They differ by the presence of a hydrophobic cavity in the CTE fold, which encloses a nonproteinaceous density of unknown identity that may be involved in giving rise to this fold. Besides AD, the Alzheimer tau fold also characterizes primary age-related tauopathy, familial British dementia, familial Danish dementia, and some prion protein amyloidoses (31, 32). The CTE tau fold is also characteristic of subacute sclerosing panencephalitis (SSPE), which is a fatal disorder of the central nervous system that is a rare consequence of infection with measles virus and begins after a symptom-free period of several years (33, 34). Tau inclusions in SSPE are also enriched in cortical layers II/III (35). Here, we report that the CTE fold is typical of tau filaments extracted from brains and spinal cords of individuals with Guam and Kii ALS/PDC, suggesting that similar molecular mechanisms underlie these diseases.

## Results

### Structural Characterization of Filaments from Guam ALS/PDC.

**Fig. 1.**
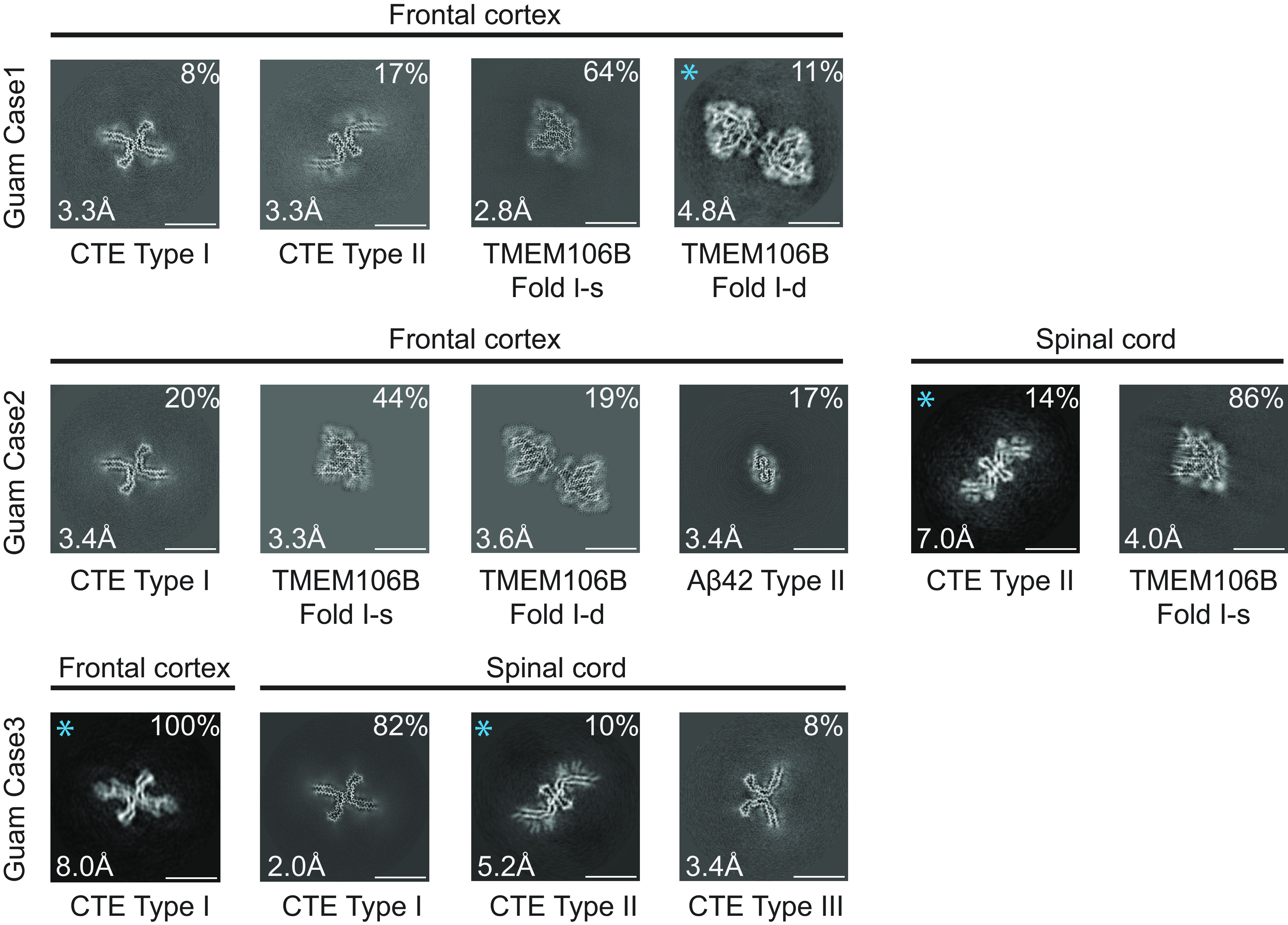
Cross-sections perpendicular to the helical axis of cryo-EM structures of filaments from the frontal cortex and spinal cord of three cases of Guam ALS/PDC, with a projected thickness of approximately one rung along the helical axis. For the filament types indicated with an asterisk, there were insufficient images for high-resolution reconstruction; identification of filament types was also based on 2D class averages. Filament types are indicated, as are structural resolutions and percentages for each type. (Scale bar, 10 nm.)

We used cryo-EM to characterize filaments from the frontal cortex of three cases of Guam ALS/PDC and the spinal cord of cases 2 and 3 (Fig. 1 and *SI Appendix*, Figs. S1–S5 and Table S1). We used optimized extraction procedures (36) to deal with the limiting amounts of brain and spinal cord samples that were available for this study (0.03 to 0.4 g per case). Staining with antitau antibody AT8 showed abundant neurofibrillary tangles (intracellular and extracellular) in frontal cortex (*SI Appendix*, Fig. S6). As described (12), tau inclusions were also found in astrocytes and oligodendrocytes, with astrocytic inclusions mostly in subpial and perivascular areas.

Tau filaments with the CTE fold were present in all cases. The frontal cortex from case 1 contained a mixture of Type I and Type II filaments, whereas that from cases 2 and 3 had only Type I filaments. The spinal cord from case 2 had only Type II filaments, whereas that from case 3 contained a mixture of Type I and Type II filaments. In addition to tau filaments, we also observed singlets and doublets of transmembrane protein 106B (TMEM106B) filaments (fold I) in the frontal cortex from cases 1 and 2, and TMEM106B singlets (fold I) in the spinal cord from case 2 (Fig. 1 and *SI Appendix*, Figs. S1 and S2). The frontal cortex from case 2 also contained Type II Aβ42 filaments (Fig. 1 and *SI Appendix*, Figs. S1 and S2), like those that were described in brain extracts from cases of AD and other diseases (36). For several filament types, there were insufficient images for de novo three-dimensional reconstruction to high resolution. Their identification was based on 2D class averages (Fig. 1 and *SI Appendix*, Fig. S1).

**Fig. 2.**
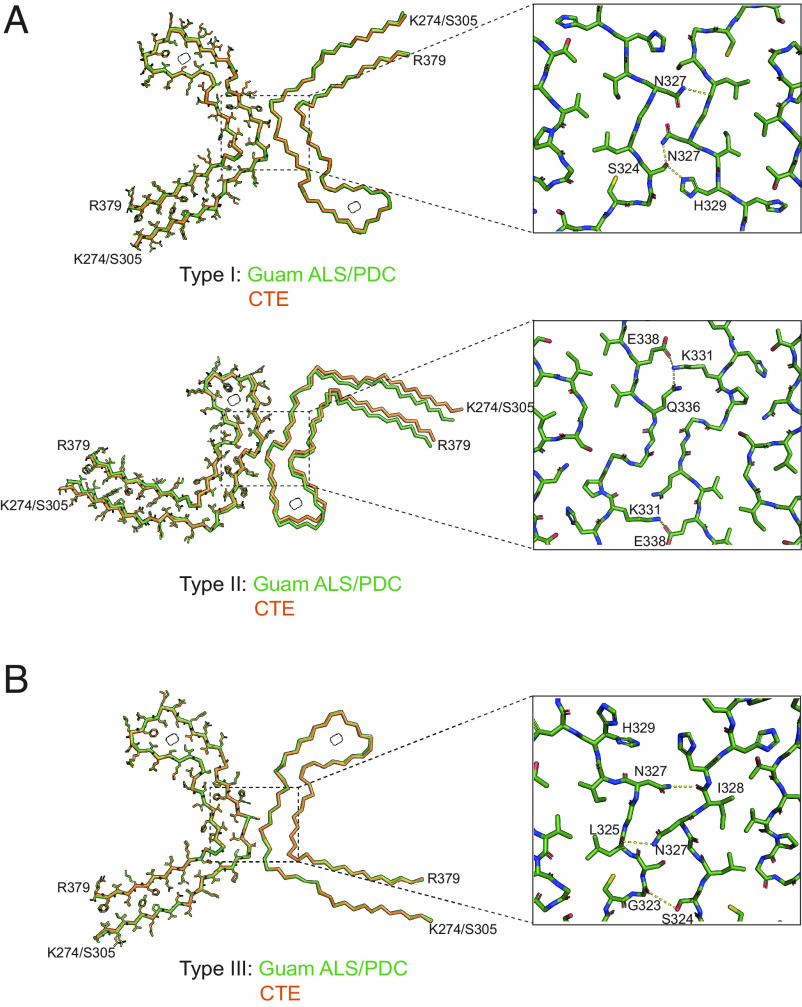
Comparison of tau filaments from Guam ALS/PDC and CTE. (*A*) Overlays of CTE Type I filaments (*Top*) and CTE Type II filaments (*Bottom*) from Guam ALS/PDC (green) and CTE (orange), with one protofilament shown in all atoms and the other protofilament shown as backbone. *Insets*: Zoomed-in views of the protofilament interfaces of both types of filaments. (*B*) Overlay of CTE Type III filaments from Guam ALS/PDC (green) and CTE (orange), shown as in (*A*). *Inset*: Zoomed-in view of the protofilament interface of CTE Type III filaments.

High-resolution structure determination confirmed that the tau filament structures from Guam ALS/PDC are identical to those from CTE (Fig. 2*A*). The rmsd of Cα atoms in one rung of the filaments between Type I filaments from the spinal cord of Guam case 3 and those from CTE (PDB:6NWP) was 0.28 Å; the rmsd between Type II filaments from the frontal cortex of Guam case 1 and those from CTE (PDB:6NWQ) was 1.36 Å.

In the spinal cord of Guam case 3, we found a small proportion of filaments (less than 10%) with a previously unobserved structure, which we named CTE Type III tau filaments (Figs. 1 and 2*B* and *SI Appendix*, Figs. S2 and S5). Like CTE Type I and Type II filaments, Type III filaments consist of two protofilaments with the CTE fold, spanning residues K274–R379 of three-repeat tau and S305–R379 of four-repeat tau, and harboring an additional density in the β-helix region. The mirror-like arrangement of protofilaments in the cross-section indicates that they adopt opposite polarities in the filaments, unlike Type I and Type II filaments. The protofilament interface consists of residues ^323^GSLGNIH^329^ from both protofilaments, like in the CTE Type I filament interface. However, they form a different, staggered parallel zipper, in which the side chains of S324 and N327 of both protofilaments intercalate and form hydrogen bonds with the main chain groups of opposite protofilaments (Fig. 2*B*). CTE Type III tau filaments were also found in a new, bigger cryo-EM dataset of filaments from the temporal cortex of an individual with CTE (case 2 in ref. 30) (*SI Appendix*, Fig. S5), indicating that they are not restricted to Guam ALS/PDC. Their 2.7 Å resolution structure revealed two alternative conformations of the side chain of H329 in one of the two protofilaments; in one conformation, its imidazole group comes into contact with the imidazole group of H329 from the opposite protofilament (Fig. 2*B* and *SI Appendix*, Fig. S5*B*). The low abundance of CTE Type III tau filaments may explain why they were not observed in the smaller cryo-EM datasets of other samples from ALS/PDC.

### Structural Characterisation of Filaments from Kii ALS/PDC.

**Fig. 3.**
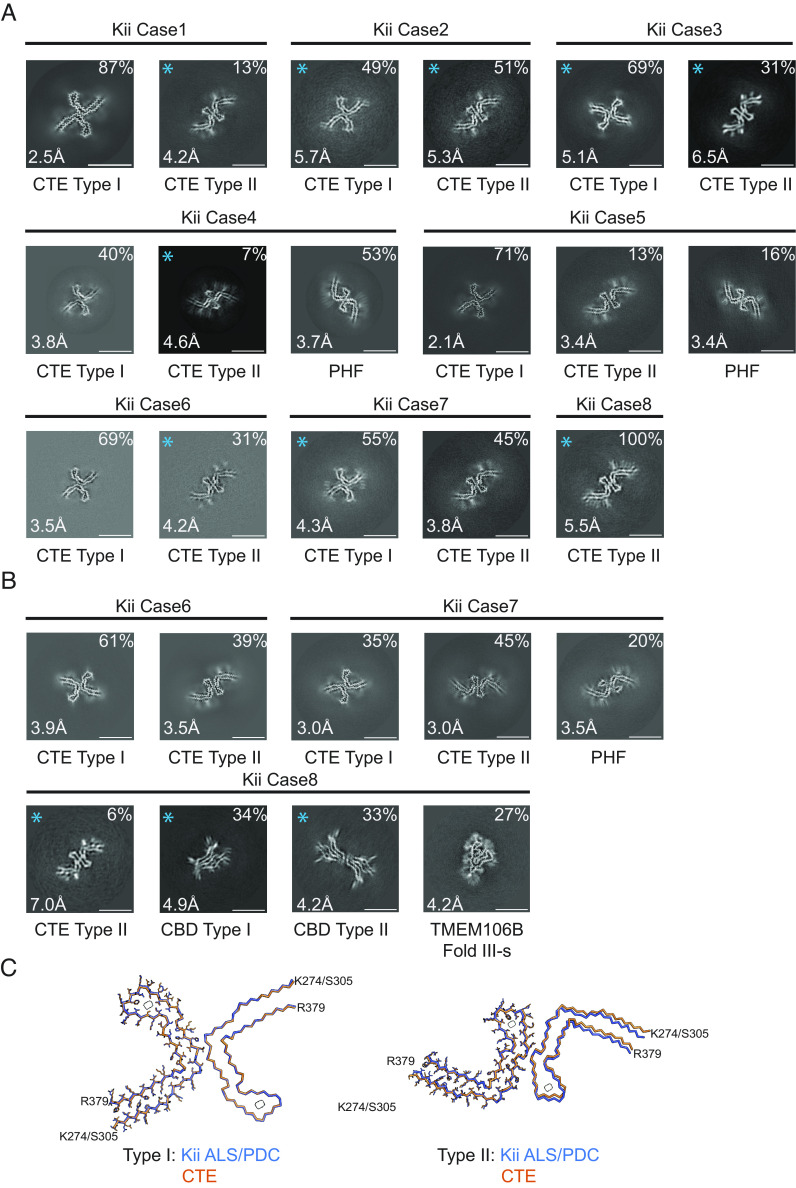
Cross-sections of cryo-EM structures of filaments from Kii ALS/PDC and comparison of tau filaments with those from CTE. (*A*) Cross-sections perpendicular to the helical axis of cryo-EM structures of filaments from eight cases of Kii ALS/PDC, with a projected thickness of approximately one rung along the helical axis. (*B*) Cross-sections of filaments from Kii ALS/PDC cases 6, 7, and 8, using the optimised extraction protocol. For the filament types indicated with an asterisk, there were insufficient images for high-resolution reconstruction; identification of filament types was also based on 2D class averages. Filament types are indicated, as are structural resolutions and percentages for each type. (Scale bar, 10 nm.) (*C*) Overlay of CTE Type I filaments (*Left*) and Type II filaments (*Right*) from Kii ALS/PDC (blue) and CTE (orange), shown as in Fig. 2.

We analyzed extracts from temporal cortex of eight cases of ALS/PDC from the Kii peninsula (Fig. 3 and *SI Appendix*, Figs. S1–S4 and Table S1). We used the standard tau filament extraction method (37) for all eight cases, and the optimised procedure (36) also for cases 6 to 8. Staining with AT8 showed the presence of abundant neurofibrillary tangles that were particularly abundant in cortical layers II/III (*SI Appendix*, Fig. S7). Tau-positive astrocytes and coiled bodies were also present. Case 8 has previously been shown to exhibit astrocytic plaque-like structures and threads, reminiscent of corticobasal degeneration (11).

Using the standard extraction method, all eight samples from the Kii peninsula contained tau filaments with the CTE fold. Case 8 had only Type II filaments; all other cases had a mixture of Type I and Type II filaments. Cases 4 and 5 also contained tau-paired helical filaments (PHFs), like those from AD and other conditions (28, 29, 31, 32). We did not observe Aβ filaments in any of the Kii cases. High-resolution structure determination showed that the structures of tau filaments from Kii ALS/PDC are also identical to those from CTE (Fig. 3*B*). The rmsd between Type I filaments from Kii case 2 and those from CTE (PDB:6NWP) was 0.38 Å; the rmsd between Type II filaments from Kii case 2 and those from CTE (PDB:6NWQ) was 1.37 Å.

Using the optimized extraction procedures that were also used for the Guam cases (36), in addition to the filament types described above, we observed tau PHFs for case 7 and TMEM106B Fold III-s filaments for case 8. In agreement with ref. 11, we also observed filaments reminiscent of α-synuclein filaments from Lewy bodies (38) for case 6 (*SI Appendix*, Fig. S1). These filaments did not show an observable twist, and we were not able to determine their three-dimensional structure. We also observed Corticobasal degeneration (CBD) Type I and CBD Type II tau filaments for case 8 (Fig. 3), consistent with the presence of astrocytic plaque-like structures (11, 39, 40).

## Discussion

Abundant filamentous amyloid inclusions that are made of all six brain tau isoforms are characteristic of ALS/PDC (8, 13). Immunoblotting of sarkosyl-insoluble tau from some of the Kii cases used here has been described previously (6, 13). We now show that tau filaments from Guam and Kii ALS/PDC adopt the CTE fold (30) in brain and spinal cord. We recently showed that tau filaments from SSPE also adopt the CTE fold (33).

The observation that specific tau filament folds characterise different diseases suggests that filament structure may provide a handle to study disease (41). For example, different cellular environments may lead to the formation of distinct structures. The observation that filaments of ALS/PDC are identical to those from CTE and SSPE thus suggests that filaments may form under similar circumstances in these diseases. It follows that the molecular mechanisms that cause tau assembly in ALS/PDC may be similar to those at work in CTE and SSPE. The latter two are probably caused by environmental factors, in the form of repetitive head injuries and measles infection, respectively. Neuroinflammation may be important in both diseases. Exogenous factors may also be causal in Guam and Kii ALS/PDC, with a possible role for parasitic infestation (21, 22).

As in CTE (26) and SSPE (35), more filamentous tau inclusions in ALS/PDC of Guam and Kii are found in layers II/III of the cerebral cortex than in layers V/VI (10, 11). This is unlike AD, where tau inclusions are more abundant in layers V/VI (25). The presence of Alzheimer and CTE tau folds correlates with these differences. It suggests that the CTE fold may also form in other diseases with a predominance of tau inclusions in cortical layers II/III that are believed to be caused by environmental factors, such as postencephalitic parkinsonism (42) and nodding syndrome (43).

The CTE tau fold differs from the Alzheimer fold by having a more open conformation of the β-helix region, which contains an internal density of unknown identity (30). In the presence of NaCl, recombinant tau comprising residues 297 to 391 assembled into filaments with the CTE fold, but in its absence, the Alzheimer tau fold formed (44). It remains to be seen how this difference relates to human brains.

Besides tau filaments with the CTE fold, we also observed tau PHFs in three cases from the Kii peninsula and tau filaments with the CBD fold in one case. Type II A β42 filaments were present in one case from Guam. Senile plaques have been described in around 60% of cases of Guam ALS/PDC (15) and assembly of Aβ is believed to be part of the disease process (45). Alternatively, these changes may be age-related. This was probably also the reason for the presence of TMEM106B filaments (46, 47) in two cases from Guam and one case from the Kii peninsula. It is possible that Aβ and TMEM106B filaments were lost during the extraction method used for some of the Kii cases. In addition to tau, also Aβ, α-synuclein and TDP-43 inclusions have been implicated in the pathogenesis of ALS/PDC (11, 14, 15). We found filaments that were reminiscent of α-synuclein filaments in one of the Kii cases. We did not observe TDP-43 filaments in any of the cases from Guam or the Kii peninsula. However, our results with two different extraction protocols for three of the Kii cases illustrate that it would be imprudent to conclude that certain filament types are not present in a brain if they are not observed with a given extraction method.

The greater heterogeneity of amyloid filaments, when compared to CTE and SSPE, probably reflects the fact that ALS/PDC is a multiple proteinopathy based on neuropathology, as suggested for cases from the Kii peninsula (11).

In conclusion, we demonstrate the presence of tau filaments with the CTE fold in cases of ALS/PDC from the island of Guam and the Kii peninsula. Type I and/or Type II CTE filaments were present in brains and spinal cords. We also describe the new CTE Type III tau filament, in which two protofilaments pack with opposite polarities. The presence of tau filaments with the CTE fold supports the hypothesis that ALS/PDC is caused by exogenous factors.

## Materials and Methods

### Cases of ALS/PDC.

Three cases of ALS/PDC from the island of Guam and eight cases from the Kii peninsula were investigated (*SI Appendix*, Table S1). The Guam cases have not been reported before; we used tissues from two Chamorro males and one ½ Chamorro, ½ Filippina female with long-standing dementia and Parkinson’s disease, in the absence of a family history of disease. They belonged to the PDC subtype, where some tau inclusions can be found in the spinal cord (48). They died aged 68 (case 1) and aged 73 (cases 2 and 3). The cases from the Kii peninsula have been published (11). Three individuals (cases 1, 2, and 5) belonged to the ALS subtype and five (cases 3, 4, and 6–8) to the PDC subtype. The ages at death were ALS subtype, 63, 76, and 77 y; PDC subtype, 60, 70, 71, 74, and 74 y. The duration of illness varied between 1 and 14 y. There was no history of head injury or measles infection in either the Guam or the Kii cases of ALS/PDC. This study was approved by the Ethics Committees of the Universities of Shinshu (3233 and 5108), Niigata (2020–0019), and Mie (2592).

### Immunohistochemistry.

Brains were fixed in 20% buffered formalin, cut into coronal sections, and paraffin-embedded. Sections (4.5 µm) were incubated overnight at room temperature with antibody AT8, which is specific for pS202 and pT205 tau (1:5,000, Innogenetics) (49). To reveal the signal, the Envision plus kit (Dako) was used, with diaminobenzidine tetrahydrochloride (Sigma-Aldrich) as chromogen. Some sections from Kii cases of ALS/PDC were also stained with Gallyas-Braak silver (50).

### Filament Extraction.

For the Guam ALS/PDC cases, we used an optimized extraction procedure, which allowed us to handle small amounts of brain samples (36). Sarkosyl-insoluble material was extracted from the frontal cortex (cases 1 to 3) and spinal cord (cases 2 and 3). The tissues (less than 400 mg) were homogenized in 3 mL buffer A (10 mM Tris-HCl, pH 7.5, 0.8 M NaCl, 10% sucrose, and 1 mM EGTA), brought to 2% sarkosyl, and incubated for 30 min at 37 °C. The samples were centrifuged at 7,000 g for 10 min, followed by spinning the supernatants at 100,000 g for 60 min. The pellets were resuspended in 100 μL/g of buffer B (20 mM Tris-HCl, pH 7.4, 100 mM NaCl) for cryo-EM analysis.

For all eight ALS/PDC cases from the Kii peninsula, filaments were extracted using the standard tau extraction method (37), with minor modifications. After incubation in 2% sarkosyl, the samples were sonicated (TAITEC ultrasonic homogeniser VP-55, level 7) for 15 s and, following a 10 min centrifugation at 27,000 g, supernatants were centrifuged at 257,400 g for 30 min at 25 °C. The pellets were then resuspended in 900 μL/g buffer A with 1% sarkosyl and centrifuged at 166,000 g for 20 min at 25 °C. For cases 6, 7, and 8 from the Kii peninsula, filaments were also extracted using the optimized procedure used for the Guam cases (36). Filaments from the CTE brain (case 2 in ref. 30) were extracted using a procedure that was developed for the extraction of TDP-43 filaments, as described (51).

### Electron Cryo-Microscopy.

Three microliters of the sarkosyl-insoluble fractions were applied to glow-discharged (Edwards S150B) holey carbon grids (Quantifoil Au R1.2/1.3, 300 mesh) that were plunge-frozen in liquid ethane using a Vitrobot Mark IV (Thermo Fisher Scientific) at 100% humidity and 4 °C. Cryo-EM images were collected on a Titan Krios electron microscope operated at 300 kV and equipped with a Falcon-4 or a K3 direct electron detector. Images were recorded in electron event representation (EER) format (52) for Falcon-4 (6 s) and Tif format for K3 (1 s), with a total dose of 40 e/Å^2^ and a pixel size of 0.824 Å (Falcon-4) or 0.826 Å (K3).

### Helical Reconstruction.

Datasets were processed in RELION using standard helical reconstruction (53). Movie frames were gain-corrected, aligned, and dose-weighted using RELION’s own motion correction program (54). Contrast transfer function (CTF) parameters were estimated using CTFFIND4-1 (55). Filaments were picked manually. For the analysis of filament types and the generation of initial three-dimensional models, segments were extracted with a box size of 1,024 pixels and down-scaled to 256 pixels. Reference-free 2D classification was performed to discard suboptimal images and measure cross-over distances for initial model calculation using relion_helix_inimodel2d (56). For high-resolution refinement, selected segments were extracted with a box size of 400 pixels, with the original pixel size. 3D auto-refinements were performed with optimization of the helical twist and rise parameters once resolutions extended beyond 4.7 Å. To improve the resolution, Bayesian polishing and CTF refinement were performed (57). Final maps were sharpened using standard postprocessing procedures in RELION and resolution estimates were calculated based on the Fourier shell correlation (FSC) between two independently refined half-maps at 0.143 (58).

### Model Building and Refinement.

For maps with resolutions beyond 4 Å, atomic models were built manually in Coot (59), based on published structures [CTE type I, PDB:6NWP; CTE type II, PDB:6NWQ; TMEM106B fold I-s, PDB:7QVC; TMEM106B fold I-d, PDB:7QVF; Type II Aβ42, PDB:7Q4M (30, 36, 41)]. Model refinements were performed using *Servalcat* (60) and REFMAC5 (61, 62). Models were validated with MolProbity (63). Figures were prepared with ChimeraX (64) and Pymol (Schrödinger, LLC.). See *SI Appendix*, Tables S2 and S3 for further details**.**

## Supplementary Material

Appendix 01 (PDF)

## Data Availability

Cryo-EM maps have been deposited in the Electron Microscopy Data Bank (EMDB) with the following accession numbers: EMD-17171 (65), EMD-17173 (66), EMD-17174 (67), EMD-17175 (68), EMD-17176 (69), EMD-17177 (70), EMD-17178 (71), EMD-17179 (72), EMD-17180 (73), and EMD-17181 (74). Corresponding refined atomic models have been deposited in the Protein Data Bank (PDB) under the following accession numbers: 8OT6 (75), 8OTC (76), 8OT9 (77), 8OTD (78), 8OTE (79), 8OTF (80), 8OTG (81), 8OTH (82), 8OTI (83), and 8OTJ (84). Please address requests for materials to the corresponding authors.
